# Multi-queen breeding is associated with the origin of inquiline social parasitism in ants

**DOI:** 10.1038/s41598-022-17595-0

**Published:** 2022-08-29

**Authors:** Romain A. Dahan, Christian Rabeling

**Affiliations:** School of Life Sciences, Arizona State University, Tempe, AZ USA

**Keywords:** Evolution, Coevolution, Phylogenetics, Social evolution, Speciation, Evolutionary ecology

## Abstract

Social parasites exploit the brood care behavior of their hosts to raise their own offspring. Social parasites are common among eusocial Hymenoptera and exhibit a wide range of distinct life history traits in ants, bees, and wasps. In ants, obligate inquiline social parasites are workerless (or nearly-so) species that engage in lifelong interactions with their hosts, taking advantage of the existing host worker forces to reproduce and exploit host colonies’ resources. Inquiline social parasites are phylogenetically diverse with approximately 100 known species that evolved at least 40 times independently in ants. Importantly, ant inquilines tend to be closely related to their hosts, an observation referred to as ‘Emery’s Rule’. Polygyny, the presence of multiple egg-laying queens, was repeatedly suggested to be associated with the origin of inquiline social parasitism, either by providing the opportunity for reproductive cheating, thereby facilitating the origin of social parasite species, and/or by making polygynous species more vulnerable to social parasitism via the acceptance of additional egg-laying queens in their colonies. Although the association between host polygyny and the evolution of social parasitism has been repeatedly discussed in the literature, it has not been statistically tested in a phylogenetic framework across the ants. Here, we conduct a meta-analysis of ant social structure and social parasitism, testing for an association between polygyny and inquiline social parasitism with a phylogenetic correction for independent evolutionary events. We find an imperfect but significant over-representation of polygynous species among hosts of inquiline social parasites, suggesting that while polygyny is not required for the maintenance of inquiline social parasitism, it (or factors associated with it) may favor the origin of socially parasitic behavior. Our results are consistent with an intra-specific origin model for the evolution of inquiline social parasites by sympatric speciation but cannot exclude the alternative, inter-specific allopatric speciation model. The diversity of social parasite behaviors and host colony structures further supports the notion that inquiline social parasites evolved in parallel across unrelated ant genera in the formicoid clade via independent evolutionary pathways.

## Introduction

Both vertebrate and invertebrate species have been the subject of many studies investigating the role of ecology in the evolution of social structure, and how variation in social systems can lead to major evolutionary transitions^1–4^. Among eusocial insects, ancestral lifetime monogamy provided the key conditions favoring the origin of eusociality across Hymenoptera^5–7^, and the ants share a single common ancestor suggesting that eusociality evolved once during the Cretaceous^8^. However, many extant ant species display social structures deviating from a single, monandrous queen in a colony^3,9,10^. While polyandry (multiple mating by females) and polygyny (multi-queen breeding) are expected to reduce intra-colonial relatedness^11^, these social structures may be beneficial to colonies. In some cases, however, when nestmate queens are highly related, polygyny can have little to no detectable effect on colony relatedness^12^. In eusocial animals, polygyny and polyandry can provide fitness benefits to the colony as a whole^11,13–15^. On the other hand, divergences in social structure in ant colonies may also result in the evolution of alternative life-history strategies, such as alternative dispersal morphs, co-operative colony foundation, reproductive cheating, and nepotism^11,16–19^.

Multi-queen nesting (polygyny) can evolve as a response to rapidly changing ecological conditions, and can have profound impacts on the social evolution of ant species^11,20–25^. In eusocial insects, polygyny may be primary, resulting from the cooperative foundation of colonies by multiple queens (i.e., pleometrosis), or secondary, resulting from the adoption of new queens in established colonies^26^. While primary polygyny is generally associated with pleometrotic colony founding under ecological conditions that promote crowding of foundress queens, and has been observed relatively rarely^11,27,28^, secondary polygyny has been a particular focus of research studying the evolutionary consequences of variation in social structure, because it provides avenues for the evolution of alternative life-history strategies^1,29,30^, and it is thought to be the prevalent mechanism for polygyny in ants^11,26^. Secondary polygyny has also repeatedly been associated with the evolution of socially parasitic life-histories in ants^31–33^.

Social parasites are species that take advantage of the social structure of their eusocial hosts to benefit their direct fitness. Three main life-histories of social parasitism occur in ants ^26,32,34^. Temporary social parasites found colonies by invading host nests, killing the host queen(s), and taking advantage of the remaining host workers to raise their first brood. Dulotic species similarly found colonies as temporary social parasites by invading host nests, but subsequently rely on frequent raids on neighboring colonies for new workers. Finally, inquiline social parasites are predominantly queen-tolerant workerless or nearly-workerless ant species which infiltrate established host colonies and take advantage of the present worker force to rear their own sexual brood^26^.

Inquiline social parasites are of particular interest to evolutionary biology because they are phylogenetically highly diverse. Inquiline (workerless) social parasitism has evolved independently at least 40 times across 25 genera in the formicoid clade of the ant tree of life^34^, and there are currently 96 known species of inquiline social parasites, which includes species arising from secondary speciation events in social parasite clades following the evolution of the parasitic life history. Empirical studies revealed that many inquiline social parasites are closely related to their hosts, a pattern known as ‘Emery’s rule’^33,35^. Thus, models describing the evolutionary origin of social parasitism must account for the rule and explain why this pattern exists. Two models have been proposed to explain the origin of inquiline social parasitism in ants. The inter-specific origin model proposes that social parasites originated as facultatively parasitic lineages of a species closely related to the incipient host(s). In contrast, the intra-specific origin model proposes that inquiline social parasites originated directly as cheating lineages from the incipient host species via sympatric speciation^26,31–33,36^. In all cases, parasites rely on similar resources, social cues, and environmental conditions as their hosts, requiring them to be fairly closely related to their hosts^33^. In the intra-specific model, hosts and parasites must necessarily form a monophyletic clade *at the time of speciation*, providing a strict explanation for why parasites and hosts tend to be more closely related than any two species within a genus^35,36^. On the other hand, the inter-specific model must include a free-living non-host species as sister to the parasite species, and cannot account for a strict interpretation of Emery’s rule. So far, empirical phylogenetic studies supported the intra-specific route of inquiline social parasite evolution in *Acromyrmex*, *Ectatomma*, *Mycocepurus*, and *Myrmica* ants^37–42^. The inter-specific model has so far garnered more support in *Pseudomyrmex* and *Temnothorax* ants^36,43^. Secondary speciation events of host and/or parasite species and host shifts can obscure the original transition to inquiline social parasitism given enough time, and lead to ambiguous resolutions between models, for example in *Pogonomyrmex*, *Solenopsis*, and a clade of Malagasy *Pheidole*^44–46^.

To explain the evolutionary transition from a cooperative eusocial lifestyle to a socially parasitic life history, secondary polygyny has repeatedly been suggested as one of the key factors^26,31–33^ because: (1) less efficient nestmate and brood recognition in polygynous colonies may yield a greater non-nestmate tolerance^47–49^; (2) the presence of multiple queens in colonies may provide an opportunity for supernumerary queens to cheat, focusing on the production of sexual offspring without contributing to the colony’s sterile worker force^30^; (3) workers and queens of polygynous colonies tolerate supernumerary egg-laying individuals^50^; and (4) the queen adoption behavior of established, secondary polygynous colonies may provide a nest-invasion ‘channel’ for social parasites to exploit^32^. These predictions have different, yet not mutually exclusive implications regarding the evolutionary dynamics of inquiline social parasitism. The former two predictions are at least partially required for intra-specific cheating to evolve, and support a sympatric, within-species origin model of inquiline social parasitism. On the other hand, the latter two predictions are required for the maintenance of current inquiline social parasitism, targeting specific host behaviors associated with secondary polygyny, and do not necessarily favor either speciation model for the origin of inquiline social parasitism. While a strong association between polygyny and inquiline social parasitism would lend support to any one of these predictions equally, a weak correlation would fail to support the latter two, as it would show that polygyny is not necessarily required for the maintenance of inquiline social parasitism. Indeed, secondary evolutionary events, after speciation of a parasite species, such as diversification or extinction events of either host or parasite species, or host shifts, are expected to obscure the evolutionary origins of social parasites and weaken an association between polygyny and inquiline social parasitism. The social structure of social parasite species is less of a focus for an intra-specific model because its main relevance would lie in the phylogenetic signal of either mono- or polygyny of their ancestors. Similarly, while multiple mating by queens (polyandry) can reduce relatedness within a colony, its occurrence would not account for cheating queen lineages in ancestors of host–parasite pairs in an intra-specific model. Furthermore, polygyny and multiple mating rarely co-occur in ants^51^. Thus, while multiple mating may play a role in the emergence of cheating, and/or parasitism in an inter-specific scenario, it is not a part of the sympatric, intra-specific model for the evolution of inquiline social parasitism per se.

The predicted association between polygyny and inquiline social parasitism has been addressed in numerous empirical studies and literature reviews^29,33,52^, however, it was never tested in a statistically rigorous framework. Here, we aim to provide a formal statistical test of the prediction that polygyny and inquiline social parasitism are associated, by conducting a meta-analysis of the social structure of ants, and performing a phylogenetically corrected test of independence between social structure and parasitism. The role of both facultative polygyny and obligate polygyny have been discussed in previous reviews, with contradicting predictions^29,33^. Thus, we further test whether obligate or facultative secondary polygyny are associated with social parasitism, in order to test whether either social structure may favor social parasitism more than the other^29,33^. Finally, we discuss our results to assess the plausibility of the four hypotheses linking polygyny and the evolution of social parasitism (as outlined above).

## Methods

### Social structure in ants

We compiled a dataset of ants with known social structures using previously published reviews as a starting point, and complemented the sampling with a thorough literature search adding species and updating information on taxonomy and social structure of species (Table S1)^1,9,13,26,29,53–55^. For each species in the dataset, we confirmed its social structure (monogyny/facultative polygyny/obligate polygyny) by researching the primary literature reference(s). As in Rissing and Pollock^55^, we validated a species as monogynous if at least 5 colonies were collected in the field. Records based on colonies that were solely lab-reared were discarded, as well as records only reported in literature reviews. Consequently, our species list does not include some of the species that were reported in previous studies, such as Keller and Passera^56^ or Keller and Reeve^53^. Every species was checked individually to ensure that the most up-to-date information was recorded. Species with known primary polygyny were excluded, as the intra-specific model predicts that secondary polygyny is associated with inquiline social parasitism. We also excluded all social parasites, because we specifically tested predictions about the social organization of the free-living hosts. In total, our final dataset comprised 331 species. In spite of our efforts, we do not claim this to be a comprehensive list of all ants with known social structure.

There is a possibility that lineages reported as monogynous in the literature might be “false negatives”, i.e., lineages were inferred as monogynous because polygynous colonies were not observed. It is impossible to prove that a species is obligately monogynous, so a certain probability of false negative species can be expected. In contrast, falsely claiming a species as polygynous appears less likely, as a polygyny inference relies on positive observations of colonies with multiple queens, and therefore, false positives are much less probable in our dataset. False negatives might bias an analysis by adding erroneous data points in the “monogynous/non-host” group, simply because of the much larger sampling in the “non-host” category. We attempted to curb this effect by stringently curating the dataset obtained from previous studies. Thus, we rejected any species where the mating biology was not directly referenced and addressed in the primary literature, as well as species where the colony structure was only inferred from lab colonies.

Some ant species practice “serial” polygyny, that is the adoption of new queens by colonies to replace an old queen^29,57^. While rarely documented, the question arises regarding the categorization of these species as either functionally monogynous or polygynous. Here, we chose to characterize them as polygynous, because the mechanism of adopting new queens still provides the opportunity for parasites to infiltrate the nest. Furthermore, some inquiline species have been shown to target old, queenless colonies of their hosts, which might originate from such a mechanism^58,59^. Similarly, species with known population-based differences in social structure (where some populations are strictly monogynous while others are either facultatively or obligately polygynous) were classified as facultatively polygynous in our dataset. Some authors further differentiate polygyny and oligogyny, where only a small percentage of the colonies are actually polygynous. We decided against this delimitation, as it seemed to introduce an arbitrariness to our data with unclear biological meaning.

### Hosts of inquiline social parasites

Similar to our survey of social structure, we collected information on hosts of inquiline social parasites from the literature, by searching for the known hosts of inquiline social parasite species. In these cases, we only retained species for which we had social structure information as well, resulting in 51 host species in the final dataset (Table 1, Table S2).

**Table 1:** Contingency table of social structure (polygyny or monogyny) in species hosting (or not hosting) obligate inquiline social parasites, before phylogenetic correction.

	Host species	Non-host species
Monogyny	9	127
	17.6%	45.4%
Polygyny	42	153
	82.4%	54.6%

Percentages represent the proportion of host and non-host species that are polygynous or monogynous.

### Phylogenetic inference and correction

In order to correct for phylogenetic non-independence, we assembled a cladogram of as many ant species as possible from available published molecular phylogenies (Table S3). We did so by “grafting” genus-level phylogenies together within subfamilies, and then “transplanting” these subfamily topologies to the appropriate tip of a subfamily-level phylogeny of Formicidae. Thus, while the branch length information was lost in the grafting, the topology of the resulting cladogram remained an accurate representation of the phylogenetic relationships between the species included. If a genus lacked an appropriate species-level phylogeny in the literature, the genus was treated as representing a hard polytomy. We discarded any species present in the phylogeny but not in the social structure dataset, and vice versa, resulting in a final dataset mapped to a cladogram of 294 species, including 48 host species of inquiline social parasites. In some cases, we had to discard phylogenies which included social parasites, because they did not include information about the social structure of host or non-host species. We then inferred ancestral states of social structure through this cladogram using the ‘ace’ function in the *ape* package for R, with default Maximum Likelihood optimization method. We inferred the social structure at a node as the one with the highest posterior probability. This procedure allowed for tracking the number of independent evolutionary transitions between social structures along the cladogram. In contrast, we inferred transitions to becoming a host for inquiline social parasites only if all of the descendants of a node were hosts, unless a case of host shift could reasonably be inferred, e.g. based on the paraphyly of a host clade relative to the parasite (see Supplementary Methods). Because this method counts evolutionary transitions toward hosting social parasites, cases in which species are hosts to multiple social parasites were only counted as a single evolutionary transition. As a result, we recovered a contingency table of independent evolutionary transitions inferred for both social structure and susceptibility to inquiline social parasites, following methods in Refs.^60,61^. For example, if a transition to becoming a host happened on a branch which started from a node inferred as polygynous, such event was counted in the Host × Polygynous cell of the contingency table. Inversely, if a reversal to monogyny was inferred on a branch whose parent node was an inferred host, or if a branch that included a transition to host had a monogynous parent node, we counted these in the Host × Monogyny cell. This method is identical to the procedure described in Schmid-Hempel and Crozier^13^.

### Statistical methods

We analyzed the results to test two hypotheses: (i) that hosts of social parasites are more likely to be polygynous compared to non-hosts; and (ii) that either facultative or obligate polygyny is overrepresented among hosts of social parasites. We tested the former by testing for the independence of social structure (monogyny vs. polygyny) from hosting (or not hosting) an inquiline social parasite. We used a one-tailed Fisher’s exact test, as the intra-specific model predicts a directional association *a priori* (i.e., that host and polygyny should be associated; as opposed to either a lack of association, or a preponderance of host/monogyny association). In contrast, we tested the second hypothesis that either obligate or facultative polygyny are involved in the evolution of inquiline social parasitism by testing for overrepresentation of either facultative or obligate polygynous species among hosts and non-hosts. Because this hypothesis lacks an *a priori* directional prediction, and the sample size was sufficiently large, we used a χ^2^ test of independence. For all tests, a significance level of 0.05 was used. All analyses were conducted in R 3.4.3, using the packages ‘ape’ ‘phytools’ and ‘base’^62–64^.

## Results

### Social structure in ants

In total, our final dataset comprised 331 species, including 51 hosts of inquiline social parasites (15.41%). Of the total 331 species, 136 (41.09%) were inferred as monogynous, while 119 (35.95%) were facultative polygynous, 28 (8.46%) were obligate polygynous, and 48 (14.50%) were inferred as polygynous without specifying whether polygyny was obligate or facultative (Tables 1, 2, Table S1). Among the 51 hosts, 42 (82.35%) were polygynous, compared to 153 polygynous species among the 280 non-host species (54.64%). Twenty-nine host species (56.86%) were facultatively polygynous, 2 (3.92%) were obligately polygynous, and 11 (21.57%) polygynous species had no reference to any social polymorphism (Table 2). After matching the dataset to our assembled cladogram, the sampling was reduced to 272 species spanning 11 subfamilies, consisting of 111 monogynous species and 161 polygynous species. Of these 272 species that were represented in the cladogram and for which reliable information on social organization was available, 47 (17.27%) were hosts of inquiline social parasites. After estimating the evolutionary history of social structure in ants, we recovered 78 transitions from monogyny to polygyny, as well as 34 reversals to monogyny (Table 3).

**Table 2:** Contingency table of polygyny type (facultative or obligate) in species hosting (or not hosting) obligate inquiline social parasites, before phylogenetic correction.

	Host species	Non-host species
Facultative polygyny	29	90
	93.6%	77.6%
Obligate polygyny	2	26
	6.4%	22.4%

Polygynous species for which information regarding obligate or facultative nature of polygyny was missing were excluded from this table. Percentages represent the proportion of host and non-host species that are facultatively or obligately polygynous.

**Table 3:** Contingency table of social structure (polygyny or monogyny) in species hosting (or not hosting) obligate inquiline social parasites, after phylogenetic correction.

	Host species	Non-host species
Monogyny background	8	26
	(12.45)	(21.55)
Polygyny background	33	45
	(28.55)	(49.45)

Numbers represent independent evolutionary events. Expected values are given in parentheses.

### Association between polygyny and social parasitism

We found a significant association between polygyny and hosting inquiline social parasites (Fisher’s exact test: Odds ratio = 0.423, *p* = 0.0443, Table 3), indicating that polygynous species were significantly over-represented among hosts of inquiline social parasites. Specifically, hosts of social parasites were more than twice as likely to be polygynous compared to non-hosts. In contrast, we did not find a significant association between either obligate or facultative secondary polygyny and social parasitism (χ^2^ test of independence: χ^2^_1_ = 3.0115, *p* = 0.0827, Table 4).

**Table 4:** Contingency table of polygyny type (facultative or obligate) in species hosting (or not hosting) obligate inquiline social parasites, after phylogenetic correction.

	Host species	Non-host species
Facultative polygyny	24	24
	(21.52)	(26.48)
Obligate polygyny	2	8
	(4.48)	(5.52)

Numbers represent independent evolutionary events. Expected values are given in parentheses.

## Discussion

We conducted a meta-analysis across the ant tree of life to statistically test for an association between social structure and being a host of inquiline social parasites, with a phylogenetic correction for independent evolutionary events. We found that polygynous species are over-represented among hosts of inquilines, confirming a long-standing but hitherto untested observation in the social parasitism literature^29,31–33^. In contrast to previous conflicting predictions^29,33^, we did not find a significant association between either obligate or facultative polygyny and inquilinism.

### Polygyny and inquiline social parasitism

The association of polygyny with social parasitism is important for both the intra-specific and the inter-specific origin models of social parasite speciation, especially relating to the tolerance of non-nestmate individuals and the queen adoption mechanism of polygynous hosts. However, while the role of multi-queen nesting is circumstantial in the inter-specific origin model (as parasites may target monogynous species as well, see below), it is critical in an intra-specific model, in which parasites evolve directly from intra-specific lineages. A sympatric origin requires polygyny in the incipient stages of the speciation process, as it would begin as reproductive cheating between nestmate queens. Here, we found a significant association between polygyny and inquiline social parasitism. While the inferred association is not definitive evidence of the intra-specific origin model, our results add to the mounting phylogenetic evidence favoring the sympatric, intra-specific speciation model for the origin of inquiline social parasites in distantly related ant lineages^38–41^. Furthermore, these observations are consistent with predictions of mechanistic models for sympatric speciation (see below)^33,65,66^. Overall, our results emphasize the importance of polygyny across ant species in the initial emergence of inquiline social parasitism, facilitating the emergence of cheating and the adoption of additional queens either intra- or inter-specifically. It is important to note that while the association generally supports an intra-specific model, it does not permit excluding the alternative, inter-specific hypothesis, and individual, taxon-specific analyses are needed to determine the evolutionary route of inquiline social parasitism in convergently evolved lineages ^34^.

### Monogyny and inquiline social parasitism

The occurrence of monogynous hosts of inquiline social parasites is at first glance inconsistent with the previously outlined arguments that polygyny promotes the evolution of inquiline social parasitism^33,52^. Nonetheless, empirical studies revealed that several social parasites have monogynous host species including, for example, inquiline species in the genera *Acromyrmex*, *Nylanderia*, and *Pogonomyrmex*^45,67,68^. To distinguish between the different evolutionary dynamics of social parasite speciation, i.e., the origin and the maintenance of social parasitism in a host population, it is important to understand whether hosts of social parasites were monogynous at the time when the social parasite originated, or whether host monogyny could be a consequence of a co-evolutionary arms-race between host and parasite^69,70^. Arms race dynamics are known to affect the social organization of the host species, and population studies of dulotic *Temnothorax* ants revealed that the frequency of monogynous colonies increased in highly parasitized host populations, presumably as a co-evolutionary response to parasitism which allowed for improved parasite detection and rejection by the host^71,72^. In general, secondary evolutionary events in either host or parasite, including host shifts, speciation and extinction events, as well as changes in social structure of either host or parasite colony, may obscure the original conditions under which social parasitism originated. If our results are correct, at least eight independent origins of inquiline social parasitism occurred in a monogynous background (Table 3), suggesting that this specialized parasitic life history can evolve in a monogynous background. Further studies are necessary to evaluate whether these eight monogynous events truly represent social parasites that evolved in monogynous hosts and whether host monogyny reflects the social colony organization at the time of parasite speciation. Once primarily monogynous hosts can be validated, it will be insightful to study the interactions and evolutionary dynamics between host and parasite species. Investigating potential natural history traits or common ecological niches in these systems may provide insights towards elucidating the evolutionary mechanisms of inquiline social parasitism in monogynous species, and the convergence of a socially parasitic life history.

### Queen size polymorphism and inquiline social parasitism

Social structure and reproductive ecology are at the core of the evolution of inquiline social parasitism^32^. Increasingly, however, other traits in the inquiline syndrome (sensu Wilson^73^) are believed to play important roles in the emergence of reproductive cheating. Queen polymorphisms involving differential dispersal strategies, for example, have provided a promising avenue of research for the investigation of intra-specific reproductive cheating^33,74–76^. Small queen morphs, referred to as ‘microgynes’, are in many species associated with alternative dispersal strategies and polygyny itself, and in some cases microgynes were shown to favor the production of sexual offspring over workers, providing the basis for reproductive cheating^30^. In some cases, microgynes are in fact considered intra-specific inquiline parasites^66,77^, whereas in other cases microgynous forms were raised to the species level as obligate inter-specific inquiline parasites^78,79^. Queen size polymorphism is heavily associated with the evolution of social parasitism based on two major lines of evidence. First, size reduction is a trait observed in most inquiline social parasites, and part of the inquiline syndrome^32,33,73,80^. Second, body size is known to affect developmental trajectory at the larval stages^75,81,82^. From these observations, it has been hypothesized that a shift of the size threshold for queen development, which would result in microgyne morphs, may also be associated with reproductive cheating. In such a case, larvae that were allocated with a certain quantity and quality of resources which would usually lead them toward worker development would instead develop into queens (the so-called ‘selfish brood hypothesis’^65,75^). From there, selection might favor the assortative mating of selfish lineages, resulting in genetic divergence and eventually speciation, resulting from alternative adaptations^33,83^. Natural history observations reinforce this second line of evidence, as some inquiline species are known to produce a worker caste, and also display a less extreme degree of size reduction compared to their hosts (e.g. the leaf-cutting ant parasite *Acromyrmex insinuator*^84^). It is important to note that the appearance and maintenance of these small queen morphs may also be adaptive to the colonies producing them, as they may evolve as dispersal or polygynous morphs in ecologically saturated habitats with limited space for new colony founding and nest establishment^17,22,85^. Further investigations of possible sources of reproductive isolation may contribute to a better understanding of how inquiline species originate from cheating lineages within their hosts^38,40,66,86^.

### Alternative evolutionary trajectories to inquiline social parasitism

In contrast to inquiline species that originated as reproductive cheaters, other ant social parasite species likely followed alternative evolutionary routes to inquilinism and workerlessness. In the formicine genus *Nylanderia*, for example, the hosts of inquiline species may be strictly monogynous^54,67,87^, possibly indicating that the inquiline social parasites originated via a different evolutionary model. Across eusocial Hymenoptera, Emery’s rule is only strictly observed in a few inquiline social parasites whereas many non-inquiline ant social parasites as well as parasitic bees and wasps are not the closest relatives of their hosts^34,35,88^. These diverse phylogenetic relationships between hosts and parasites suggest that social cheating evolved convergently along independent pathways in convergently evolved host parasite systems. In at least two different systems, inquiline social parasites are nested in clades featuring other socially parasitic life histories. For example, molecular phylogenies suggest that the socially parasitic *Temnothorax corsicus* species group, a clade formerly known as *Myrmoxenus*^43,89^, which contains primarily dulotic species, also contains a number of independently evolved so-called ‘murder-parasites’ or ‘degenerate dulotic’ species, which are workerless or nearly-workerless species that display morphological and behavioral traits generally associated with inquiline social parasitism^90^. These origins are thought to likely emerge as a loss of the worker caste in dulotic species and demonstrate that some inquiline social parasites may evolve from a dulotic ancestor^91^. Similarly, *Formica talbotae*, the only confirmed inquiline species in the wood ant genus *Formica*, likely evolved from a temporary social parasitic ancestor in the *F. difficilis* group (the former *F. microgyna* group)^26,92–94^. In both cases, an intra-specific origin of social parasitism is unlikely, and these species provide evidence for inquiline social parasites evolving from either a dulotic or a temporary social parasitic ancestor instead of a free-living, polygynous ancestor. Furthermore, these *Temnothorax* and *Formica* social parasite species suggest a potential for evolutionary transitions between socially parasitic life history strategies^10,26,32,34,52^.

### Limitations of the study

Considering the current knowledge about the phylogenetic relationships and the social structure of individual ant species, we chose a conservative approach to test whether polygyny is statistically associated with being a host of an inquiline social parasite. Nonetheless, we would like to point out some limitations of our study that arise from uncertainties at different levels of the data collection and analysis, and therefore, future studies may come to a different conclusion. The results of this study are not invalidated by these shortcomings, and will hopefully be addressed in the future:

#### Species selection

We assembled a comprehensive and carefully curated dataset of ant species with known social structure. Nonetheless, the social organization and natural history is only known for a small subset (371 species in our dataset) of the world’s ant species (14,035 recognized species), representing only 2.4%. Therefore, our inference may change as new information on the social structure of additional ant species becomes available. Furthermore, we applied a conservative filter to our species selection, only retaining social organization data for species with documented social structures that were observed in nature. We chose to discard information about the social structure of species which were solely reported from laboratory-bred colonies.

#### Phylogenetic inference

Ideally, this study would have included a high-quality phylogeny inferred from a comprehensive molecular genetic dataset. This would have allowed us to include statistical measures of confidence for the topology (i.e., bootstrap values or Bayesian posterior probabilities), as well as branch length information for a more refined ancestral character state inference. Considering the different datasets (i.e., morphological vs mitochondrial and nuclear genetic vs genomic and transcriptomic data) that have been utilized over the past decades to infer phylogenies across diverse ant taxa, assembling a data matrix and inferring a comprehensive phylogeny spanning the taxa analyzed in this this study was not feasible. Therefore, we decided to construct a cladogram by grafting trees from previously published phylogenies that were inferred from diverse data matrices (Table S2). By applying this methodology, we retained only the topological information from these phylogenies representing the relationships between the species in our dataset, but discarding branch length information and discarding estimates of confidence regarding the topology. Once comprehensive genetic/genomic data will become available for the entire ant tree of life, a high-quality phylogeny should be used to quantify how uncertainty at the level of tree inference affects the conclusion of our study.

#### Ancestral character state inference

Because of the method we used to assemble the cladogram, a metric tree was not available to perform an ancestral character state estimation using Maximum Likelihood or Bayesian methods. In the absence of branch lengths, and by analyzing discrete characters, our analysis relied on a maximum parsimony approach, which assumes that transitions from monogyny to polygyny and their reversal (from polygyny to monogyny) are equally likely on each edge of the tree. Future studies need to evaluate whether transitions from monogyny to polygyny and vice versa are in fact equally likely.

## Conclusion

The comparative analysis presented here reveals a significant association between secondary polygyny and being a host of an inquiline social parasite in ants, and becoming a host of a social parasite appears to be twice as likely in a polygynous species. However, this association is imperfect, in that it affects species with obligate or facultative polygyny equally. Our results support the previously formulated hypothesis that polygyny is an important trait associated with the evolutionary origins of inquiline social parasitism in ants. However, secondary evolutionary transitions such as host shifts, speciation and extinction events, as well as changes in colony organization in either the host or parasite may erode the signal of the original condition under which social parasitism originated. Furthermore, co-evolutionary arms race dynamics between host and parasite may result in secondarily monogynous hosts of inquiline social parasites. Considering that obligate inquiline social parasitism has evolved in parallel many times in distantly related clades across the ant tree of life, it is unrealistic to expect that every convergent origin of the inquiline phenotype evolved along identical trajectories. We discussed potential avenues to investigate and contrast different mechanisms through which obligate workerless inquiline parasitism might have evolved in ants, both in sympatry and in allopatry. Overall, our results emphasize that changes in social structure can have significant consequences for life history and social evolution in eusocial insects.

## Supplementary Information

Supplementary Information 1.

Supplementary Information 2.

Supplementary Information 3.

## Data Availability

All data is included in the supplementary material as well as on Dryad data repository, and is available at the following link: https://datadryad.org/stash/share/KgQrS5tgVtXzz6SU8jcYB0w4CMBe7I-FGLYL1GFZ4d4.
