# Supplementary material for: Multi-queen breeding is associated with the origin of inquiline social parasitism in ants
Source: Sci Rep. 2022 Aug 29;12:14680. doi: 10.1038/s41598-022-17595-0 (PMC9424252; doi:10.1038/s41598-022-17595-0)
Supplement: Supplementary file 2 — Supplementary Information 2. [file 41598_2022_17595_MOESM2_ESM.docx]

**Title: Multi-queen breeding is associated with the origin of inquiline social parasitism in ants**

**Supplementary Material**

*Romain A. Dahan and Christian Rabeling*

*School of Life Sciences, Arizona State University, Tempe AZ, USA*

Correspondence: Romain A. Dahan (romain.a.dahan@gmail.com), Christian Rabeling ([crabeling@gmail.com](mailto:crabeling@gmail.com))

| **Species** | **Social**  **structure** | **Host.non.host** | **Type** | **in.phylogeny** | **Evidence** | **Reference** |
| --- | --- | --- | --- | --- | --- | --- |
| *Acromyrmex_ambiguus* | P | Host | F | y | I | Dahan et al. 2022 |
| *Acromyrmex_crassispinus* | P | Host | F | y | I | Dahan et al. 2022 |
| *Acromyrmex_echinatior* | P | Host | F | y | I | Bekkevold et al. 1999 |
| *Acromyrmex_heyeri* | P | Host | F | y | I | Diehl et al. 2001; Dahan et al. 2022 |
| *Acromyrmex_landolti* | M | Non-host | NA | y | I | Keller & Reeve 1994 |
| *Acromyrmex_lobicornis* | M | Non-host | NA | y | I | Dahan et al. 2022 |
| *Acromyrmex_lundii* | M | Host | NA | y | I | Dahan et al. 2022 |
| *Acromyrmex_octospinosus* | M | Host | NA | y | I | Boomsma et al 1999 |
| *Acromyrmex_subterraneus* | P | Host | U | y | II | De Souza et al. 2004; 2005 |
| *Anoplolepis_gracilipes* | P | Non-host | O | y | VI | Drescher et al. 2007 |
| *Aphaenogaster_cockerelli* | M | Non-host | NA | y | VI | Hölldobler & Carlin 1989 |
| *Aphaenogaster_japonica* | M | Non-host | NA | y | III | Mizutani & Immamura 1980 |
| *Aphaenogaster_rudis* | P | Non-host | F | y | III | Boulay et al. 2007 |
| *Aphaenogaster_senilis* | M | Non-host | NA | y | III | Cheron et al. 2009; Boulay et al. 2007 |
| *Aphaenogaster_subterranea* | M | Non-host | NA | y | V | Stukalyuk & Radchenko 2011 |
| *Aphaenogaster_treatae* | M | Non-host | NA | y | III | Talbot 1954 |
| *Apterostigma_dentigerum* | M | Non-host | NA | y | II | Forsyth 1981 |
| *Apterostigma_mayri* | M | Non-host | NA | y | III | Murakami et al. 2000 |
| *Atta_colombica* | M | Non-host | NA | y | I | Fjerdingstad et al. 1998 |
| *Atta_sexdens* | M | Non-host | NA | y | I | Fjerdingstad & Boomsma 2000 |
| *Azteca_longiceps* | M | Non-host | NA | y | VI | Frumhoff & Ward 1992 |
| *Brachymyrmex_depilis* | M | Non-host | NA | y | III | Keller & Reeve 1994 |
| *Camponotus_americanus* | M | Non-host | NA | n | VI | Frumhoff & Ward 1992 |
| *Camponotus_consobrinus* | P | Non-host | F | n | I | Fraser et al. 2000 |
| *Camponotus_festinatus* | P | Non-host | F | y | I | Goodisman & Hahn 2005 |
| *Camponotus_floridanus* | M | Non-host | NA | y | VI | Frumhoff & Ward 1992 |
| *Camponotus_herculaneus* | P | Non-host | U | n | VI | H_lldobler 1961 |
| *Camponotus_impressus* | M | Non-host | NA | n | VI | Frumhoff & Ward 1992 |
| *Camponotus_ligniperda* | P | Non-host | U | n | VI | H_lldobler 1961 |
| *Camponotus_nawai* | M | Non-host | NA | n | III | Satoh 1989; 1991 |
| *Camponotus_nearcticus* | M | Non-host | NA | y | VI | Frumhoff & Ward 1992 |
| *Camponotus_novaeboracensis* | M | Non-host | NA | y | VI | Frumhoff & Ward 1992 |
| *Camponotus_ocreatus* | M | Non-host | NA | y | III | Goodisman & Hahn 2004 |
| *Camponotus_pennsylvanicus* | M | Non-host | NA | y | III | Pricer 1908 |
| *Camponotus_planatus* | P | Non-host | U | n | VI | Frumhoff & Ward 1992 |
| *Camponotus_subbarbatus* | M | Non-host | NA | n | VI | Frumhoff & Ward 1992 |
| *Camponotus_tortuganus* | M | Non-host | NA | n | VI | Frumhoff & Ward 1992 |
| *Camponotus_werthi* | M | Non-host | NA | n | III | Skaife 1961 |
| *Camponotus_yamaokai* | P | Non-host | O | n | III | Terayama & Satoh 1990; Satoh 1989 |
| *Cardiocondyla_atalanta* | P | Non-host | F | y | V | Heinze et al. 2005 |
| *Cardiocondyla_batesii* | M | Non-host | NA | y | V | Heinze et al. 2005 |
| *Cardiocondyla_elegans* | M | Non-host | NA | y | V | Heinze et al. 2005 |
| *Cardiocondyla_emeryi* | P | Non-host | F | y | V | Heinze et al. 2005 |
| *Cardiocondyla_kagutsuchi* | P | Non-host | F | y | V | Heinze et al. 2005 |
| *Cardiocondyla_mauritanica* | P | Non-host | F | y | V | Heinze et al. 2005 |
| *Cardiocondyla_minutior* | P | Non-host | F | y | V | Heinze et al. 2005 |
| *Cardiocondyla_nigra* | M | Non-host | NA | y | V | Heinze et al. 2005 |
| *Cardiocondyla_nuda* | P | Non-host | U | y | VI | Frumhoff & Ward 1992 |
| *Cardiocondyla_obscurior* | P | Non-host | F | y | V | Heinze et al. 2005 |
| *Cardiocondyla_shuckardi* | P | Host | U | y | III | Heinze et al 2014 |
| *Cardiocondyla_strigifrons* | P | Non-host | F | n | V | Heinze et al. 2005 |
| *Cardiocondyla_ulianini* | M | Non-host | NA | y | V | Heinze et al. 2005 |
| *Cardiocondyla_wroughtonii* | P | Non-host | F | y | V | Heinze et al. 2005 |
| *Cataglyphis_bicolor* | M | Host | NA | y | VI | Wehner et al. 1994 |
| *Cataglyphis_bombycinus* | M | Non-host | NA | y | I | Leniaud et al. 2013 |
| *Cataglyphis_emmae* | M | Non-host | NA | n | I | Jowers et al. 2013 |
| *Cataglyphis_hispanica* | M | Non-host | NA | y | I | Leniaud et al. 2012 |
| *Cataglyphis_livida* | M | Non-host | NA | n | I | Timmermans et al. 2010 |
| *Cataglyphis_mauritanica* | P | Non-host | O | y | I | Eyer et al 2013 |
| *Cataglyphis_niger* | P | Non-host | U | y | I | Leniaud et al. 2011 |
| *Cataglyphis_sabulosa* | M | Non-host | NA | n | I | Timmermans et al. 2008 |
| *Cataglyphis_savigny* | M | Non-host | NA | n | I | Leniaud et al 2011 |
| *Cataglyphis_theryi* | M | Non-host | NA | n | I | Leniaud et al 2013 |
| *Cataglyphis_velox* | P | Non-host | F | y | I | Eyer et al 2013 |
| *Cephalotes_atratus* | M | Non-host | NA | y | VI | Corn 1980 |
| *Cephalotes_minutus* | M | Non-host | NA | y | VI | Frumhoff & Ward 1992 |
| *Cephalotes_setulifer* | M | Non-host | NA | y | VI | Frumhoff & Ward 1992 |
| *Colobopsis_nipponica* | M | Non-host | NA | y | VI(I) | Hasegawa 1994 |
| *Crematogaster_abstinens* | M | Non-host | NA |  | III | Martins Segundo et al. 2017 |
| *Crematogaster_ashmeadi* | M | Non-host | NA | y | VI | Frumhoff & Ward 1992 |
| *Crematogaster_atkinsoni* | P | Non-host | U | y | VI | Frumhoff & Ward 1992 |
| *Crematogaster_biroi* | M | Non-host | NA | y | III | Peeters et al. 2013 |
| *Crematogaster_carinata* | P | Non-host | O | y | V | Longino 2003 |
| *Crematogaster_cerasi* | M | Non-host | NA | y | VI | Frumhoff & Ward 1992 |
| *Crematogaster_crinosa* | P | Non-host | F | y | V | Longino 2003 |
| *Crematogaster_emeryana* | M | Non-host | NA | y | VI | Frumhoff & Ward 1992 |
| *Crematogaster_erecta* | P | Non-host | F | y | V | Longino 2003 |
| *Crematogaster_gerstaeckeri* | P | Non-host | U | y | VI | Stanton et al 2002 |
| *Crematogaster_laeviuscula* | M | Non-host | NA | y | VI | Frumhoff & Ward 1992 |
| *Crematogaster_limata* | P | Non-host | F | y | V | Longino 2003 |
| *Crematogaster_lineolata* | M | Non-host | NA | y | VI | Frumhoff & Ward 1992 |
| *Crematogaster_longispina* | P | Non-host | U | y | V | Longino 2003 |
| *Crematogaster_marioni* | M | Non-host | NA | y | VI | Frumhoff & Ward 1992 |
| *Crematogaster_minutissima* | P | Non-host | U | y | VI | Frumhoff & Ward 1992 |
| *Crematogaster_missouriensis* | M | Non-host | NA | y | I | Heinze et al 2000 |
| *Crematogaster_monteverdensis* | P | Non-host | O | y | V | Longino 2003 |
| *Crematogaster_nigropilosa* | P | Non-host | F | y | V | Longino 2003 |
| *Crematogaster_pygmaea* | P | Non-host | O | y | III | Quinet et al 2009 |
| *Crematogaster_schimmeri* | M | Non-host | NA | y | III | Peeters et al. 2013 |
| *Crematogaster_scutellaris* | M | Non-host | NA | y | VI | Frumhoff & Ward 1992 |
| *Crematogaster_sumichrasti* | P | Non-host | U | y | V | Longino 2003 |
| *Cyphomyrmex_costatus* | M | Non-host | NA | y | III | Murakami et al. 2000 |
| *Cyphomyrmex_longiscapus* | M | Non-host | NA | y | III | Mueller & Wcislo 1998 |
| *Cyphomyrmex_rimosus* | P | Non-host | F | y | II | Murakami et al. 2000 |
| *Dolichoderus_mariae* | P | Non-host | O | y | III | Laskis & Tchinkel 2009 |
| *Dolichoderus_plagiatus* | P | Non-host | F | y | III | Kannowski 1967 |
| *Dolichoderus_pustulatus* | P | Non-host | F | y | III | Kannowski 1967 |
| *Dolichoderus_quadripunctatus* | M | Non-host | NA | y | III | Torossian 1960 |
| *Dorylus_nigricans_molestus* | P | Non-host | F | y | I | Kronauer et al. 2004 |
| *Dorymyrmex_bicolor* | P | Non-host | F | y | I | Berkelhamer 1984 |
| *Dorymyrmex_flavopecta* | M | Non-host | NA | y | III | Nickerson et al. 1975 |
| *Dorymyrmex_grandula* | M | Non-host | NA | y | VI | Frumhoff & Ward 1992 |
| *Dorymyrmex_insana* | M | Non-host | NA | y | I | Berkelhamer 1984; Nickerson et al 1975 |
| *Dorymyrmex_smithi* | P | Non-host | O | y | VI | Wagner & Fleur Nicklen 2006 |
| *Labidus_coecus* | M | Non-host | NA |  | III | Rettenmeyer & Watkins 1978 |
| *Eciton_burchellii* | M | Non-host | NA | y | VI | Rettenmeyer & Watkins 1978 |
| *Eciton_dulcium_crassinode* | M | Non-host | NA | y | VI | Rettenmeyer & Watkins 1978 |
| *Eciton_hamatum* | M | Non-host | NA | y | VI | Rettenmeyer & Watkins 1978 |
| *Eciton_lucanoides* | M | Non-host | NA | y | VI | Rettenmeyer & Watkins 1978 |
| *Eciton_mexicanum* | M | Non-host | NA | y | VI | Rettenmeyer & Watkins 1978 |
| *Eciton_vagans_angustatum* | M | Non-host | NA | y | VI | Rettenmeyer & Watkins 1978 |
| *Ectatomma_ruidum* | P | Non-host | F | y | III | Pratt 1989; Breed et al 1990 |
| *Ectatomma_tuberculatum* | P | Host | F | y | II | Hora et al. 2005 |
| *Forelius_pruinosus* | P | Non-host | F | y | I | Holldobler 1982; Berkelhamer 1984 |
| *Formica_accreta* | P | Non-host | U | y | VI | Frumhoff & Ward 1992 |
| *Formica_aquilonia* | P | Non-host | O | n | VI | Rosengren et al. 1993 |
| *Formica_argentea* | P | Non-host | U | n | I | Snyder 1993 |
| *Formica_cinerea* | P | Non-host | O | y | VI | Rosengren et al. 1993 |
| *Formica_exsecta* | P | Non-host | F | y | III | Pisarski 1972; 1973; Pamilo & Rosengen 1984 |
| *Formica_exsectoides* | P | Non-host | U | n | III | Scherba 1961 |
| *Formica_fusca* | P | Non-host | F | y | VI | Rosengren et al. 1993 |
| *Formica_incerta* | P | Non-host | F | y | III | Talbot 1948 |
| *Formica_japonica* | P | Non-host | F | n | II | Masuko et al 1998 |
| *Formica_lugubris* | P | Non-host | O | y | VI | Rosengren et al. 1993 |
| *Formica_montana* | P | Non-host | F | y | III | Henderson & Jeanne 1992 |
| *Formica_neogagates* | P | Non-host | U | y | VI | Frumhoff & Ward 1992 |
| *Formica_obscuripes* | P | Host | O | y | III | Finnegan 1977 |
| *Formica_obscuriventris* | M | Non-host | NA | y | VI | Frumhoff & Ward 1992 |
| *Formica_opaciventris* | P | Non-host | O | y | III | Scherba 1961 |
| *Formica_pallidefulva* | M | Non-host | NA | y | III | Talbot 1948 |
| *Formica_paralugubris* | P | Non-host | O | n | III | Chapuisat et al 1999 |
| *Formica_podzolica* | P | Non-host | F | n | III | Deslippe & Savolainen 1995 |
| *Formica_polyctena* | P | Non-host | F | y | IV | Seifert 1991; Pamilo 1982 |
| *Formica_pressilabris* | P | Non-host | U | n | III | Pamilo & Rosengren 1984 |
| *Formica_rufa* | P | Non-host | F | y | III | Seifert 1991 |
| *Formica_selysi* | P | Non-host | F | n | I | Purcell et al 2015 |
| *Formica_subsericea* | P | Non-host | U | y | VI | Frumhoff & Ward 1992 |
| *Formica_transkaucasica* | P | Non-host | F | n | III | Mabelis & Chardon 2005; Pamilo 1982 |
| *Formica_truncorum* | P | Non-host | U | y | III | Rosengren et al. 1985; Sundstrom 1993; Elias et al. 2004 |
| *Formica_ulkei* | P | Non-host | O |  | III | Scherba 1961 |
| *Formica_yessensis* | P | Non-host | O |  | III | Higashi 1983 |
| *Gnamptogenys_striatula* | P | Non-host | O | y | V | Blatrix & Jaisson 2000 |
| *Hypoponera_ergatandria* | P | Non-host | F | y | II | Yamauchi et al. 1996; Seifert 2003 |
| *Iridomyrmex_purpureus* | P | Non-host | F | y | I | Halliday 1983 |
| *Lasius_alienus* | M | Non-host | NA | y | VI | Frumhoff & Ward 1992 |
| *Lasius_flavus* | P | Non-host | F | y | I | Steinmeyer et al. 2012 |
| *Lasius_neglectus* | P | Non-host | O | y | III | Van Loon et al. 1990 |
| *Lasius_neoniger* | M | Non-host | NA | n | VI | Frumhoff & Ward 1992 |
| *Lasius_niger* | M | Non-host | NA | y | III | Gaspar 1965 |
| *Lasius_pallitarsis* | M | Non-host | NA | n | III | Nonacs 1990 |
| *Lasius_sakagamii* | P | Non-host | O | y | II | Yamauchi et al. 1981; 1982 |
| *Leptogenys_intermedia* | M | Non-host | NA | y | III | Villet et al. 1991 |
| *Leptothorax_acervorum* | P | Host | F | y | I | Douwes et al. 1987 |
| *Leptothorax_gredleri* | M | Non-host | NA | y | VI | Buschinger 1968 |
| *Leptothorax_muscorum* | P | Host | F | y | III | Henize & Buschinger 1988; Buschinger 1979 |
| *Leptothorax_retractus* | P | Non-host | F |  | III | Heinze & Buschinger 1988 |
| *Leptothorax_scamni* | M | Non-host | NA | n | II | Heinze & Gratiashvili 2015 |
| *Linepithema_humile* | P | Non-host | O | y | III | Tsutsui & Case 2001; Newell & Barber 1913 |
| *Manica_bradleyi* | P | Host | F | y | VI | Wheeler & Wheeler 1970 |
| *Manica_rubida* | P | Non-host | U | y | I | Cammaerts & Cammaerts 1987; Lenoir et al. 2010 |
| *Megalomyrmex_modestus* | P | Non-host | U | y | V | Boudinot et al. 2013 |
| *Megalomyrmex_silvestrii* | P | Non-host | F | y | V | Boudinot et al. 2013 |
| *Messor_ebeninus* | M | Non-host | NA | y | III | Tohme 1975 |
| *Monomorium_cyaneum* | P | Host | F | y | III | DuBois 2000 |
| *Monomorium_ebeninum* | P | Non-host | F | y | III | DuBois 2000 |
| *Monomorium_emarginatum* | P | Non-host | F | y | VI | Frumhoff & Ward 1992 |
| *Monomorium_ergatogyna* | P | Non-host | F | y | III | DuBois 2000 |
| *Monomorium_floricola* | P | Host | U | y | VI | Frumhoff & Ward 1992 |
| *Monomorium_minimum* | P | Host | F | y | III | DuBois 2000 |
| *Monomorium_pharaonis* | P | Non-host | O | y | III | DuBois 2000 |
| *Monomorium_salomonis* | P | Host | O | y | VI | Forel 1928 |
| *Monomorium_viridum* | P | Non-host | F | y | III | DuBois 2000 |
| *Mycetophylax_conformis* | M | Non-host | NA | y | III | Klingenberg et al. 2007 |
| *Mycetophylax_morschi* | M | Non-host | NA | y | III | Klingenberg et al. 2007 |
| *Mycetophylax_simplex* | M | Non-host | NA | n | III | Klingenberg et al. 2007 |
| *Mycocepurus_goeldii* | P | Host | F | y | I | Rabeling & Bacci 2010; Rabeling et al. in prep |
| *Myrmecia_brevinoda* | P | Non-host | F |  | I | Qian et al. 2011 |
| *Myrmecia_dispar* | M | Non-host | NA | y | IV | Gray 1971 |
| *Myrmecia_gulosa* | M | Non-host | NA | y | VI | Haskins & Haskins 1980 |
| *Myrmecia_nigriceps* | M | Non-host | NA | y | Vi | Haskins & Haskins 1980 |
| *Myrmecia_pilosula* | P | Non-host | F | y | I | Craig & Crozier 1979 |
| *Myrmecia_vindex* | M | Host | NA | y | VI | Keller & Genoud 1997; Haskins & Haskins 1980 |
| *Myrmecina_graminicola* | P | Non-host | F | y | II | Buschinger & Schreiber 2002 |
| *Myrmecina_nipponica* | P | Non-host | F | y | II | Okhawara et al. 1993 |
| *Myrmecocystus_depilis* | M | Non-host | NA | y | I | Hölldobler et al. 2011 |
| *Myrmica _obscura* | P | Non-host | F | n | VI | Radchenko & Elmes 2010 |
| *Myrmica_alaskensis* | P | Host | U | y | VI | Glasier et al. 2014 |
| *Myrmica_americana* | P | Non-host | F | y | III | Keller & Reeve 1994 |
| *Myrmica_cagnianti* | P | Host | U | n | III | Espadaler 1996 |
| *Myrmica_constricta* | P | Non-host | U | y | V | Czekes et al 2012 |
| *Myrmica_gallienii* | P | Host | F | n | I | Elmes & Petal 1990; Sepp_ 1996 |
| *Myrmica_hellenica* | P | Non-host | F | y | V | Czekes et al 2012 |
| *Myrmica_incompleta* | P | Non-host | U | y | VI | Buschinger et al. 1980; Lenoir et al. 1992 |
| *Myrmica_latifrons* | P | Non-host | F |  | III | Kannowski 1970 |
| *Myrmica_lobicornis* | P | Host | F | y | III | Kannoski 1970; Sepp_ 1994 |
| *Myrmica_lonae* | P | Host | U | y | V | Czekes et al 2012 |
| *Myrmica_pinetorum* | M | Non-host | NA | n | VI | Frumhoff & Ward 1992 |
| *Myrmica_punctiventris* | P | Non-host | F | y | III | Bansbach and Herbers 1996 |
| *Myrmica_rubra* | P | Host | F | y | I | Elmes & Petal 1990; Leppannen et al 2014 |
| *Myrmica_ruginodis* | P | Host | F | y | V | Czekes et al 2012 |
| *Myrmica_rugulosa* | P | Host | U | y | V | Czechowski 1979; Czekes et al 2012 |
| *Myrmica_sabuleti* | P | Host | F | y | I | Sepp_ 1996 |
| *Myrmica_scabrinodis* | P | Host | F | y | I | Sepp_ 1996 |
| *Myrmica_schenki* | P | Non-host | F | y | III | Elmes 1980 |
| *Myrmica_serica* | P | Non-host | F | n | V | Radchenko & Elmes 2010 |
| *Myrmica_specioides* | P | Non-host | F | n | V | Jansen & Radchenko 2009 |
| *Myrmica_spinosior* | P | Host | U | y | V | Radchenko & Elmes 2010 |
| *Myrmica_sulcinodis* | P | Non-host | F | y | III | Radchenko & Elmes 2010; Elmes 1987 |
| *Myrmica_tahoensis* | P | Non-host | F | y | I | Evans 1998 |
| *Myrmicocrypta_ednaella* | M | Non-host | NA | y | II | Murakami & Higashi 1997 |
| *Myrmica_stangeana* | P | Non-host | F | n | V | Radchenko & Elmes 2010 |
| *Neivamyrmex_carolinensis* | P | Non-host | F | y | I | Rettenmeyer & Watkins 1978; Kronauer & Boomsma 2007 |
| *Neivamyrmex_nigrescens* | M | Non-host | NA | y | III | Rettenmeyer & Watkins 1978 |
| *Neivamyrmex_opacithorax* | M | Non-host | NA | y | III | Rettenmeyer & Watkins 1978 |
| *Nothomyrmecia_macrops* | M | Non-host | NA | y | I | Sanetra & Crozier 2001 |
| *Nylanderia_arenivaga* | M | Non-host | NA | y | VI | Frumhoff & Ward 1992 |
| *Nylanderia_flavipes* | M | Non-host | NA | y | III | Ichinose 1994 |
| *Nylanderia_fulva* | P | Non-host | O | y | III | Arcila et al. 2002; McDonald 2012 |
| *Nylanderia_parvula* | M | Host | NA | y | VI | Frumhoff & Ward 1992 |
| *Odontomachus_bauri* | M | Non-host | NA | y | III | Ehmer & H_lldobler 1995 |
| *Odontomachus_cephalotes* | P | Non-host | U | y | VI | Peeters 1987; Ito et al 1996 |
| *Odontomachus_rixosus* | P | Non-host | O | y | II | Ito et al 1996 |
| *Odontomachus_ruginodis* | M | Non-host | NA | y | VI | Frumhoff & Ward 1992 |
| *Odontomachus_troglodytes* | P | Non-host | F | y | III | Colombel 1970 |
| *Oecophylla_longinoda* | M | Non-host | NA | y | III | Hölldobler & Wilson 1983 |
| *Paltothyreus_tarsatus* | M | Non-host | NA | y | III | Braun et al. 1994 |
| *Paratrechina_longicornis* | P | Non-host | U | y | III/VI | Yamauchi & Ogata 1995 |
| *Pheidole_cockerelli* | M | Non-host | NA | y | VI | Frumhoff & Ward 1992 |
| *Pheidole_desertorum* | M | Non-host | NA | y | I | Helms 1999 |
| *Pheidole_floridana* | M | Non-host | NA | y | VI | Frumhoff & Ward 1992 |
| *Pheidole_megacephala* | P | Host | U | y | VI | Hoffmann 1998 |
| *Pheidole_pallidula* | P | Non-host | F | y | I | Fournier et al 2002 |
| *Pheidole_pilifera* | M | Host | NA | y | VI | Frumhoff & Ward 1992 |
| *Pheidole_tysoni* | M | Non-host | NA | y | VI | Frumhoff & Ward 1992 |
| *Plagiolepis_pygmaea* | P | Host | F | y | III | Passera et al. 2001 |
| *Plagiolepis_vindobonensis* | P | Host | U | y | VI | Faber 1969; Buschinger 1990 |
| *Pogonomyrmex_badius* | M | Non-host | NA | y | VI | Keller & Reeve 1994 |
| *Pogonomyrmex_barbatus* | M | Host | NA | y | I | Suni et al. 2007 |
| *Pogonomyrmex_desertorum* | M | Non-host | NA | y | VI | Keller & Reeve 1994 |
| *Pogonomyrmex_maricopa* | M | Non-host | NA | y | VI | Keller & Reeve 1994 |
| *Pogonomyrmex_montanus* | M | Non-host | NA | y | III | MacKay 1981 |
| *Pogonomyrmex_occidentalis* | M | Non-host | NA | y | I | Wiernasz et al. 2004 |
| *Pogonomyrmex_pima* | P | Non-host | F | y | I | Tate Holbrook et al. 2007; Strehl 2005 |
| *Pogonomyrmex_rugosus* | M | Host | NA | y | III | Gadau et al 2002 |
| *Pogonomyrmex_subnitidus* | M | Non-host | NA | y | III | Mackay 1981 |
| *Ponera_coarctata* | P | Non-host | F | y | III | Liebig et al. 1995 |
| *Ponera_pennsylvanica* | P | Non-host | U | y | III | Pratt et al. 1994 |
| *Prenolepis_imparis* | P | Non-host | F | y | VI | Tschinkel 1987 |
| *Proatta_butelli* | P | Non-host | O | y | III | Moffet 1986 |
| *Proceratium_silaceum* | P | Non-host | U | y | VI | Frumhoff & Ward 1992 |
| *Proformica_longiseta* | P | Non-host | F | y | I | Sepp_ et al. 2008 |
| *Pseudomyrmex_apache* | P | Non-host | F | y | III | Ward 1985 |
| *Pseudomyrmex_ejectus* | P | Host | F | n | VI | Ward 1985 |
| *Pseudomyrmex_ferrugineus* | P | Non-host | F |  | V | Ward 1993 |
| *Pseudomyrmex_flavicornis* | M | Non-host | NA | y | V | Ward 1993 |
| *Pseudomyrmex_janzeni* | P | Non-host | F | n | V | Ward 1993; Janzen 1973 |
| *Pseudomyrmex_mixtecus* | M | Non-host | NA | y | V | Ward 1993 |
| *Pseudomyrmex_nigrocinctus* | M | Non-host | NA | y | V | Ward 1993 |
| *Pseudomyrmex_pallidus* | P | Host | F | y | III | Ward 1985 |
| *Pseudomyrmex_peperi* | P | Non-host | F | y | I | Kautz et al. 2009 |
| *Pseudomyrmex_satanicus* | P | Non-host | O | y | V | Ward 1993; Janzen 1974 |
| *Pseudomyrmex_seminole* | M | Non-host | NA | n | III | Ward 1985 |
| *Pseudomyrmex_simplex* | P | Non-host | F | y | IV | Ward 1985 |
| *Pseudomyrmex_spinicola* | M | Non-host | NA | y | V | Ward 1993 |
| *Pseudomyrmex_veneficus* | P | Non-host | O | y | V | Ward 1993 |
| *Rhytidoponera_chalybaea* | P | Non-host | F | y | I | Ward 1980 |
| *Rhytidoponera_confusa* | P | Non-host | F | y | I | Ward 1980 |
| *Rhytidoponera_purpurea* | P | Non-host | F | y | I | Ward 1980 |
| *Sericomyrmex_amabilis* | P | Non-host | F | y | I | Villesen et al 2002 |
| *Solenopsis_carolinensis* | P | Non-host | U | y | VI | Frumhoff & Ward 1992 |
| *Solenopsis_corticalis* | P | Non-host | U | y | V | Thompson 1989 |
| *Solenopsis_germinata* | P | Non-host | F | y | II/III | Adams et al 1976 |
| *Solenopsis_invicta* | P | Non-host | F | y | II/III | Greenberg et al 1985 |
| *Solenopsis_molesta* | P | Non-host | U | y | VI | Frumhoff & Ward 1992 |
| *Solenopsis_picta* | P | Non-host | U | y | VI | Frumhoff & Ward 1992 |
| *Solenopsis_quinquecuspis* | P | Host | F | y | II | Jouvenaz et al 1989 |
| *Solenopsis_richteri* | P | Host | F | y | II | Jouvenaz et al 1989 |
| *Solenopsis_texana* | P | Non-host | U | y | VI | Frumhoff & Ward 1992 |
| *Stenamma_brevicorne* | P | Non-host | F |  | IVb | Talbot 1974 |
| *Stenamma_debile* | P | Non-host | F | y | II | Buschinger 1999 |
| *Stenamma_diecki* | P | Non-host | F |  | IVb | Talbot 1974 |
| *Stigmatomma_pallipes* | P | Non-host | F | y | II/III | Traniello 1982 |
| *Strumigenys_gundlachi* | M | Non-host | NA | y | VI | Frumhoff & Ward 1992 |
| *Tapinoma_erraticum* | P | Host | F | y | III | Cournault & Aron 2009 |
| *Tapinoma_litorale* | P | Non-host | U | y | VI | Frumhoff & Ward 1992 |
| *Tapinoma_melanocephalum* | P | Non-host | F | y | II | Bustos & Cherix 1998 |
| *Tapinoma_minutum* | P | Non-host | F | y | IV | Herbers 1991 |
| *Tapinoma_sessile* | P | Non-host | U | y | VI | Buczkowski & Bennett 2008 |
| *Technomyrmex_albipes* | P | Non-host | U | y | II | Yamauchi et al. 1991 |
| *Temnothorax_affinis* | P | Non-host | F | n | III | Buschinger 1968 |
| *Temnothorax_allardycei* | M | Non-host | NA | y | VI | Frumhoff & Ward 1992 |
| *Temnothorax_ambiguus* | P | Non-host | F | y | III | Alloway et al 1982 |
| *Temnothorax_angustulus* | M | Non-host | NA | y | VI | Frumhoff & Ward 1992 |
| *Temnothorax_ariadnae* | M | Non-host | NA | n | III | Salata et al 2018 |
| *Temnothorax_aveli* | M | Non-host | NA | n | VI | Keller 1998 |
| *Temnothorax_carinatus* | M | Non-host | NA | y | V | MacKay 2000 |
| *Temnothorax_crassispinus* | M | Non-host | NA | n | III | Ticha & Stys 2002; Ticha 1992; Ticha 2002 |
| *Temnothorax_curvispinosus* | P | Host | F | y | III | Alloway et al. 1982 |
| *Temnothorax_exilis* | P | Host | F | y | V | Salata et al. 2018 |
| *Temnothorax_helenae* | M | Non-host | NA | n | V | Salata et al. 2018 |
| *Temnothorax_lichtensteini* | M | Non-host | NA | n | VI | Keller 1998 |
| *Temnothorax_longispinosus* | P | Non-host | F | y | III | Alloway et al 1982 |
| *Temnothorax_neomexicanus* | M | Non-host | NA | y | VI | Frumhoff & Ward 1992 |
| *Temnothorax_nigriceps* | P | Non-host | F | y | II | Buschinger 1968 |
| *Temnothorax_nylanderi* | P | Non-host | F | y | V | Buschinger 1968 |
| *Temnothorax_obliquicanthus* | M | Non-host | NA | y | VI | Frumhoff & Ward 1992 |
| *Temnothorax_obturator* | M | Non-host | NA | y | VI | Frumhoff & Ward 1992 |
| *Temnothorax_parvulus* | M | Non-host | NA | n | VI | Stukalyduk & Radchenko 2011 |
| *Temnothorax_pastinifer* | M | Non-host | NA | y | VI | Frumhoff & Ward 1992 |
| *Temnothorax_pergandei* | M | Non-host | NA | y | VI | Frumhoff & Ward 1992 |
| *Temnothorax_recedens* | P | Non-host | U | n | V | Salata et al 2018 |
| *Temnothorax_rugatulus* | P | Host | F | y | I | Rüppell et al. 1998; Rüppell et al 2002 |
| *Temnothorax_spinosior* | P | Non-host | F | y | I | Hamaguchi et al. 1993 |
| *Temnothorax_tuberum* | P | Host | F | n | II | Buschinger 1968 |
| *Temnothorax_tuscaloosae* | P | Non-host | F | y | II | Guenard et al. 2016 |
| *Temnothorax_unifasciatus* | M | Non-host | NA | y | IVb | Buschinger 1968 |
| *Tetramorium_alpestre* | P | Host | F | y | II | Steiner 2003; Wagner et al. 2017 |
| *Tetramorium_bicarinatum* | P | Non-host | U | y | VI | Astruc et al. 2001 |
| *Tetramorium_caespitum* | M | Host | NA | y | V | Wagner et al. 2017 |
| *Tetramorium_caldarium* | P | Non-host | U | y | VI | Frumhoff & Ward 1992 |
| *Tetramorium_impurum* | P | Host | F | y | VI | Steiner et al 2003; Buschinger 1974 |
| *Tetramorium_moravicum* | P | Non-host | F | y | VI | Shlick-Steiner et al. 2005 |
| *Tetramorium_simillimum* | P | Non-host | U | y | VI | Frumhoff & Ward 1992 |
| *Tetraponera_anthracina* | P | Non-host | F | y | III | Terron 1977 |
| *Trachymyrmex_isthmicus* | M | Non-host | NA | y | III | Murakami et al. 2000 |
| *Trachymyrmex_septentrionalis* | M | Non-host | NA | y | VI | Frumhoff & Ward 1992 |
| *Trachymyrmex_turrifex* | M | Non-host | NA | y | V | Rabeling et al. 2007 |
| *Vollenhovia_emeryi* | P | Host | U | y | VI | Kinomura & Yamauchi 1994; Okamoto & Ohkawara 2010 |
| *Wasmannia_auropunctata* | P | Non-host | O | y | V | Ulloa-Chacon & Cherix 1988 |
| *Xenomyrmex_floridanus* | M | Non-host | NA | y | VI | Frumhoff & Ward 1992 |
| *Leptanella_japonica* | M | Non-host | NA | y | III | Masuko 1990 |

**Table S1:** List of the species curated in the dataset and their reported social structures. The social structure is given as M (monogynous) and P (polygynous). The “Type” column refers to whether polygyny is obligate (O), facultative (F), or not specified as either one (U). The evidence categories are summarized in table S4.

| **Host** | **Parasite** |
| --- | --- |
| *Acromyrmex ambiguus* | *Pseudoatta argentina* |
| *Acromyrmex crassispinus* | *Pseudoatta argentina* |
| *Acromyrmex echinatior* | *Acromyrmex insinuator* |
| *Acromyrmex heyeri* | *Acromyrmex charruanus, Pseudoatta argentina* |
| *Acromyrmex lundii* | *Pseudoatta argentina* |
| *Acromyrmex octospinosus* | *Acromyrmex insinuator* |
| *Acromyrmex subterraneus* | *Acromyrmex ameliae* |
| *Cardiocondyla shuckardi* | *Cardiocondyla zoserska* |
| *Cataglyphis bicolor* | *Cataglyphis hanae* |
| *Ectatomma tuberculatum* | *Ectatomma parasiticum* |
| *Formica obscuripes* | *Formica talbotae* |
| *Leptothorax acervorum* | *Leptothorax goesswaldi, L. kutteri, L. pacis* |
| *Leptothorax muscorum* | *Leptothorax paraxenus, L. wilsoni* |
| *Manica bradleyi* | *Manica parasitica* |
| *Monomorium cyaneum* | *Monomorium inquilinum* |
| *Monomorium floricola* | *Monomorium inquilinum* |
| *Monomorium minimum* | *Monomorium inquilinum* |
| *Monomorium salomonis* | *Monomorium inquilinum* |
| *Mycocepurus goeldii* | *Mycocepurus castrator* |
| *Myrmecia vindex* | *Myrmecia inquilina* |
| *Myrmica alaskensis* | *Myrmica lampra, M. quebecensis* |
| *Myrmica cagnianti* | *Myrmica kabylica* |
| *Myrmica gallienii* | *Myrmica karavajevi* |
| *Myrmica lobicornis* | *Myrmica myrmicoxena* |
| *Myrmica lonae* | *Myrmica hirsuta, M. karavajevi* |
| *Myrmica rubra* | *Myrmica rubra microgyne* |
| *Myrmica ruginodis* | *Myrmica karavajevi* |
| *Myrmica rugulosa* | *Myrmica karavajevi* |
| *Myrmica sabuleti* | *Myrmica karavajevi* |
| *Myrmica scabrinodis* | *Myrmica laurae, M. karavajevi* |
| *Myrmica spinosior* | *Myrmica laurae, M. lemasnei* |
| *Nylanderia parvula* | *Nylanderia deceptrix* |
| *Pheidole megacephala* | *Pheidole neokohli* |
| *Pheidole pilifera* | *Pheidole inquilina* |
| *Plagiolepis pygmaea* | *Plagiolepis grassei, P. xene* |
| *Plagiolepis vindobonensis* | *Plagiolepis regis* |
| *Pogonomyrmex barbatus* | *Pogonomyrmex anergismus* |
| *Pogonomyrmex rugosus* | *Pogonomyrmex anergismus, P. colei* |
| *Pseudomyrmex ejectus* | *Pseudomyrmex leptosus* |
| *Pseudomyrmex pallidus* | *Pseudomyrmex inquilinus* |
| *Solenopsis quinquecuspis* | *Solenopsis daguerrei* |
| *Solenopsis richteri* | *Solenopsis daguerrei* |
| *Tapinoma erraticum* | *Tetramorium schmitzi* |
| *Temnothorax curvispinosus* | *Temnothorax minutissimus* |
| *Temnothorax exilis* | *Temnothorax corsicus* |
| *Temnothorax rugatulus* | *Temnothorax rugatulus microgyne* |
| *Temnothorax tuberum* | *Temnothorax corsicus* |
| *Tetramorium alpestre* | *Tetramorium inquilinum* |
| *Tetramorium caespitum* | *Tetramorium inquilinum, T. kutteri, T. atratulum* |
| *Tetramorium impurum* | *Tetramorium inquilinum, T. atratulum* |
| *Vollenhovia emeryi* | *Vollenhovia nipponica* |

**Table 2:** List of the host species in the dataset and their inquiline social parasites.

| **Group** | **Level** | **Source** |
| --- | --- | --- |
| Formicidae | Sub-Families | [1] |
| Ponerinae | Genera | [2] |
| Dorylinae | Genera | [3] |
| Dolichoderinae | Genera | [4] |
| Pseudomyrmecinae | Genera | [5] |
| Myrmecinae | Genera | [6] |
| Formicinae | Genera | [7] |
| Amblyoponinae | Genera | [8] |
| *Myrmecia* | Species | [9] |
| *Lasius* | Species | [10] |
| *Pseudomyrmex* | Species | [11] |
| *Myrmica* | Species | [12] |
| *Cataglyphis* | Species | [13] |
| *Formica* | Species | [14] |
| *Anochetus* | Species | [15] |
| *Odontomachus* | Species | [15] |
| *Temnothorax* | Species | [16] |

**Table S3:** Phylogenetic information and references used for the reconstruction of a cladogram. Only genera retained after filtering the data for maximum statistical power are shown. Taxa with only one species in the social structure dataset were equally omitted.

| Evidence class | Evidence |
| --- | --- |
| I | Molecular analyses |
| II | Queen dissections |
| III | >10% polygyny, or 100% monogyny among field colonies |
| IV | 5%<X<10% polygyny |
| IVb | <5% polygyny |
| V | Taxonomic monograph |
| VI | Mentioned in passing/Personal Communications |

**Table S4:** Evidence categories used in table S1.


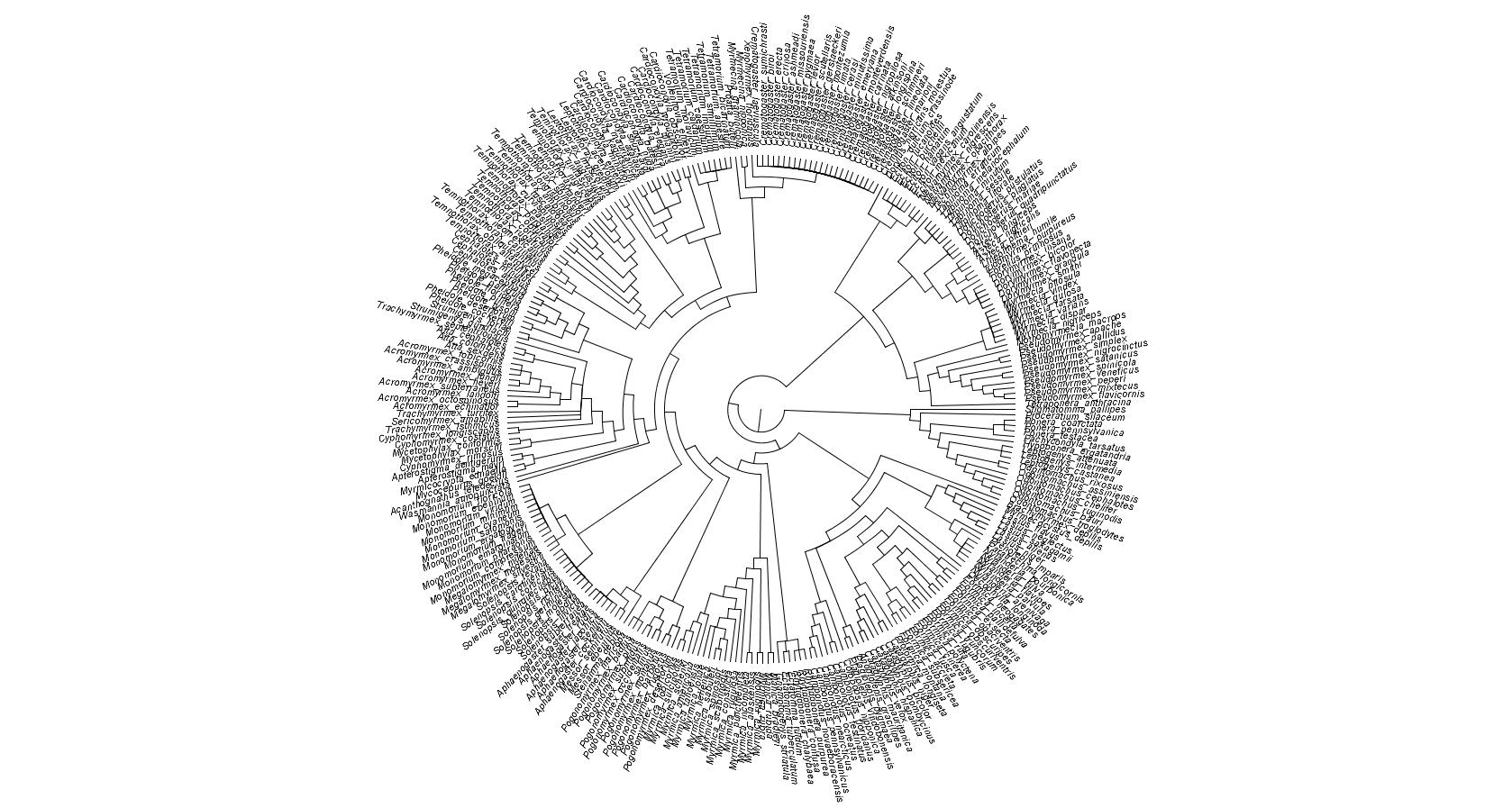


**Figure S1:** Cladogram used in the phylogenetic analyses.

**References**

1. Borowiec ML, Rabeling C, Brady SG, Fisher BL, Schultz TR, Ward PS. 2017 Compositional heterogeneity and outgroup choice influence the internal phylogeny of the ants. *bioRxiv* , 173393. (doi:10.1101/173393)

2. Schmidt CA, Shattuck SO. 2014 The Higher Classification of the Ant Subfamily Ponerinae (Hymenoptera: Formicinae), with review of Ponerinae ecology and Behavior. *Zootaxa* **3817**, 1–242. (doi:http://dx.doi.org/10.11646/zootaxa.3817.1.1)

3. Borowiec ML. 2016 Generic revision of the ant subfamily Dorylinae (hymenoptera, formicidae). *Zookeys* **608**, 1–280. (doi:10.3897/zookeys.608.9427)

4. Ward PS, Brady SG, Fisher BL, Schultz TR. 2010 Phylogeny and biogeography of dolichoderine ants: Effects of data partitioning and relict taxa on historical inference. *Syst. Biol.* **59**, 342–362. (doi:10.1093/sysbio/syq012)

5. Ward PS, Downie DA. 2005 The ant subfamily Pseudomyrmecinae (Hymenoptera: Formicidae): Phylogeny and evolution of big-eyed arboreal ants. *Syst. Entomol.* **30**, 310–335. (doi:10.1111/j.1365-3113.2004.00281.x)

6. Ward PS, Brady SG, Fisher BL, Schultz TR. 2015 The evolution of myrmicine ants: Phylogeny and biogeography of a hyperdiverse ant clade (Hymenoptera: Formicidae). *Syst. Entomol.* **40**, 61–81. (doi:10.1111/syen.12090)

7. Ward PS, Blaimer BB, Fisher BL. 2016 A revised phylogenetic classification of the ant subfamily Formicinae (Hymenoptera: Formicidae), with resurrection of the genera Colobopsis and Dinomyrmex. *Zootaxa* **4072**, 343–357. (doi:10.11646/zootaxa.4072.3.4)

8. Ward PS, Fisher BL. 2016 Tales of dracula ants: The evolutionary history of the ant subfamily Amblyoponinae (Hymenoptera: Formicidae). *Syst. Entomol.* **41**, 683–693. (doi:10.1111/syen.12186)

9. Hasegawa E, Crozier RH. 2006 Phylogenetic relationships among species groups of the ant genus *Myrmecia*. *Mol. Phylogenet. Evol.* **38**, 575–582. (doi:10.1016/j.ympev.2005.09.021)

10. Maruyama M, Steiner FM, Stauffer C, Akino T, Crozier RH, Schlick-Steiner BC. 2008 A DNA and morphology based phylogenetic framework of the ant genus *Lasius* with hypotheses for the evolution of social parasitism and fungiculture. *BMC Evol. Biol.* **8**, 1–15. (doi:10.1186/1471-2148-8-237)

11. Gómez-Acevedo S, Rico-Arce L, Delgado-Salinas A, Magallón S, Eguiarte LE. 2010 Neotropical mutualism between *Acacia* and Pseudomyrmex: Phylogeny and divergence times. *Mol. Phylogenet. Evol.* **56**, 393–408. (doi:10.1016/j.ympev.2010.03.018)

12. Jansen G, Savolainen R, Vepsäläinen K. 2010 Phylogeny, divergence-time estimation, biogeography and social parasite-host relationships of the Holarctic ant genus *Myrmica* (Hymenoptera: Formicidae). *Mol. Phylogenet. Evol.* **56**, 294–304. (doi:10.1016/j.ympev.2010.01.029)

13. Knaden M, Tinaut A, Stökl J, Cerdá X, Wehner R. 2012 Molecular phylogeny of the desert ant genus *Cataglyphis* (Hymenoptera: Formicidae). *Myrmecol. News* **16**, 123–132.

14. Blaimer BB, Brady SG, Schultz TR, Lloyd MW, Fisher BL, Ward PS. 2015 Phylogenomic methods outperform traditional multi-locus approaches in resolving deep evolutionary history: a case study of formicine ants. *BMC Evol. Biol.* **15**, 271. (doi:10.1186/s12862-015-0552-5)

15. Larabee FJ, Fisher BK, Schmidt CA, Matos-Maraví P, Janda M, Suarez A V. 2016 Molecular phylogenetics and diversification of trap-jaw ants in the genera *Anochetus* and *Odontomachus* (Hymenoptera: Formicidae). *Mol. Phylogenet. Evol.* **103**, 143–154. (doi:10.1016/j.ympev.2016.07.024)

16. Prebus M. 2017 Insights into the evolution, biogeography and natural history of the acorn ants, genus Temnothorax Mayr (hymenoptera: Formicidae). *BMC Evol. Biol.* **17**, 1–22. (doi:10.1186/s12862-017-1095-8)
